# Lipid-gated “phosphorylation code” for TCR graded signaling and T-cell exhaustion

**DOI:** 10.1093/procel/pwaf110

**Published:** 2025-12-24

**Authors:** Hui Chen, Jizhong Lou, Wei Chen

**Affiliations:** State Key Laboratory of Epigenetic Regulation and Intervention, Institute of Biophysics, Chinese Academy of Sciences, Beijing 100101, China; State Key Laboratory of Epigenetic Regulation and Intervention, Institute of Biophysics, Chinese Academy of Sciences, Beijing 100101, China; Department of Cardiology, The Second Affiliated Hospital, Zhejiang University School of Medicine, Hangzhou 310012, China; Liangzhu Laboratory, Hangzhou 310012, China

The T cell receptor (TCR)/CD3s complex represents one of the most sophisticated signaling machineries in immunology, capable of translating diverse antigenic stimulations into finely tuned cellular outcomes—ranging from activation and proliferation to differentiation and exhaustion, ultimately orchestrating T-cell fate transitions (Minguet et al., 2025). At the heart of this complex lies the CD3ζ chain, whose cytoplasmic domain contains three immunoreceptor tyrosine-based activation motifs (ITAMs). Remarkably, these ITAMs account for nearly 60% of all phosphorylatable tyrosine residues in the entire TCR/CD3s complex. Owing to its central role in propagating TCR signals, CD3ζ has also become a foundational component in chimeric antigen receptors (CAR) design, serving as the key signaling module that underpins the success of CAR-T cell therapies.

In a recent cover article in *Molecular Cell*, Xu and colleagues (Li et al., 2025) have uncovered a lipid**-**electrostatic regulatory mechanism that dictates the sequential phosphorylation of CD3ζ ITAMs. This elegant study not only fills a long-standing gap in our understanding of how TCR signaling achieves its remarkable versatility, but also reveals a new intrinsic lipid-dependent pathway contributing to T-cell exhaustion (Fig. 1). These findings provide fresh mechanistic insight into T-cell regulation and open new avenues for immunotherapeutic intervention targeting the CD3ζ module.

While cryo-electron microscopy (cryo-EM) studies have resolved the extracellular and transmembrane architectures of the TCR/CD3s complex in both ligand-bound and unbound states (Dong et al., 2019; Susac et al., 2022), the cytoplasmic domain of CD3ζ has long resisted structural characterization. Its intrinsically disorder and dynamics nature, coupled with tight association with the plasma membrane, has posed formidable technical challenges—until now.

Xu and colleagues overcame these challenges using solution nuclear magnetic resonance (NMR) spectroscopy within a bicelle-based membrane-mimetic system, which recapitulates the lipid composition and biophysical properties of the native plasma membrane. This approach enabled atomic-level analysis of the CD3ζ cytoplasmic domain, revealing a striking gradient of membrane insertion across its three ITAMs (Fig. 1A). Specifically, ITAM1 (N-terminal) exhibited shallow embedding with high solvent accessibility, ITAM2 adopted an intermediate insertion balancing membrane association and solvent exposure, whereas ITAM3 (C-terminal) penetrated deeply into the hydrophobic core of the membrane with minimal solvent contact.

Critical to this gradient is not the primary sequence of the ITAMs themselves, but rather electrostatic interactions between adjacent basic residue rich sequences (BRSs) in CD3ζ and acidic phospholipids in the membrane. Substitution of key arginine residues in these BRSs disrupted the gradient, confirming BRS-lipid binding as the driving force of the structural heterogeneity.

These findings further reinforce a growing conceptual model in TCR biology: CD3 cytoplasmic tails are not fully disordered but adopt conditionally ordered conformations upon membrane association. Similar to CD3ε, whose cytoplasmic domain undergoes lipid-dependent manner to sequester its ITAM (Prakaash et al., 2021; Xu et al., 2008), CD3ζ uses membrane binding as a conserved regulatory mechanism to modulate ITAM accessibility and find-tune TCR signaling initiation, ultimately preventing spurious TCR activation.

To link structural heterogeneity to functional outcome, Xu and colleagues employed targeted quantitative mass spectrometry (MS) and immunoblotting to precisely map the phosphorylation kinetics of CD3ζ ITAMs. Their analyses uncovered a strict N-to-C terminal phosphorylation hierarchy: ITAM1 was phosphorylated most rapidly, followed by ITAM2, and finally ITAM3. This order directly correlates with the membrane insertion gradient, whereby the shallowly embedded ITAM1 is the most readily accessible to the Src-family kinase Lck, while deeply buried ITAM3 requires greater energetic input for changing its modification (Fig. 1A).

Crucially, this hierarchical pattern depends on both the membrane environment and the integrity of BRS–lipid interactions. Replacing acidic phospholipids with neutral phosphatidylcholine eliminates the insertion gradient, leading to unordered ITAM phosphorylation. Similarly, disrupting BRS-lipid interactions via BRS mutation also abolishes the insertion gradient and sequential phosphorylation. These results establish BRS–lipid electrostatic association as a central regulatory mechanism in TCR signaling initiation, complementary to previously described interactions between BRSs and the Src unique domain, as well as ITAMs and the Src kinase domain (Chen et al., 2023; Li et al., 2017; Shah et al., 2018). Together, these multi-layered interactions encode a “phosphorylation code,” transforming the TCR from a binary on/off switch into a finely tunable signaling apparatus, as previously suggested for CD3ε (Wu et al., 2020).

This elegant study provides a long-sought explanation for the graded nature of TCR signaling. Rather than responding in an all-or-nothing fashion, the TCR functions as a tunable signal integrator. Antagonistic antigens (e.g., self-peptides, weak tumor antigens with low-affinity and/or slip-bond binding with TCR) may only trigger phosphorylation of the most accessible ITAM1, generating basal tonic signals necessary for T cell survival without full activation (Qin et al., 2025). In contrast, agonistic antigens (e.g., strong pathogen-derived agonist peptides forming higher binding affinity and catch-bond with TCR) drive optimal coreceptor cooperation and Lck rapid activation, leading to complete phosphorylation across all ITAMs for eliciting robust T-cell activation, cytokine production, and effector differentiation (Hong et al., 2018; Liu et al., 2014; Qin et al., 2025; Wu et al., 2019).

The mechano-regulation of TCR antigen recognition appears to be closely linked to this graded nature of TCR signaling. In our previous study, T cell simulation with varying agonistic antigens revealed that differential TCR signaling is triggered by cooperatively effects of varying amplitudes of mechanical forces and force-dependent dissociation kinetics (Liu et al., 2014). This different force-dependent TCR triggering may associate with the ITAM phosphorylation hierarchy, that is, three ITAMs phosphorylation may sequentially triggered by mechanical force for producing phosphorylation hierarchy. This phenomenon extends beyond TCR signaling. Multiple receptors and signaling molecules exhibit graded activation in response to varying mechanical stimuli. For example, BCR activation displays similarly graded activation in response to mechanical stimulation (Wan et al., 2018), and Piezo1 channels undergo distinct conformational changes that lead to graded gating (Xiao, 2024). It is thus plausible that the graded activation of other receptors may also be linked to the outside-in mechanical regulation. While direct evidence is currently lacking, this represents an interesting and plausible mechanistic direction that warrants further investigation.

This graded signaling model provides a compelling explanation for how individual T cells fine-tune their responses to antigens of varying strength—a fundamental mechanism for maintaining the delicate balance between immunity and tolerance. Indeed, the functional importance of ITAM diversity has long been recognized in T cell development (Bettini et al., 2017). Intriguingly, insights from CAR-T cell engineering further underscore this principle. Sadelain and colleagues showed that selectively deleting either the distal or proximal two ITAMs within the CD3ζ chain significantly enhanced the anti-tumor efficacy of 1928z CAR-T cells (Feucht et al., 2019), highlighting that ITAM-specific phosphorylation patterns can directly dictate T cell functional outcomes. These insights open exciting avenues for next-generation synthetic receptor design. For example, engineering BRS sequences to impose tailored phosphorylation hierarchies or to optimize signaling strength and duration, potentially enabling the rational tuning of T-cell responses. This new strategy could offer a powerful framework for improving immunotherapy precision and durability.

Perhaps the most intriguing and clinically impactful finding of this study is the identification of an intrinsic pathway of TCR signal attenuation that contributes to T cell exhaustion. T cell exhaustion is a state of dysfunction characterized by impaired proliferation, reduced cytokine production, and upregulation of inhibitory receptors such as PD-1, CTLA-4, Tim-3, Lag3. This state commonly arises under conditions of chronic antigen exposure, such as persistent infection or within the tumor microenvironment. To mimic chronic stimulation, Xu and colleagues repeatedly activated primary human T cells with anti-CD3 antibodies. Under these conditions, CD3ζ phosphorylation became progressively impaired, especially at the distal ITAM3. This led to accumulation of partially phosphorylated CD3ζ and weakened downstream signaling.

Mechanistically, the authors traced this defect to ATP limitation. Chronic stimulation may reduce intracellular ATP availability. Since phosphorylating deeply membrane-embedded ITAM3 requires more energetic input than that of ITAM1, ATP depletion preferentially impaired ITAM3 modification (Fig. 1B–C). Thus, chronic stimulation favors partial rather than complete phosphorylation of CD3ζ, revealing a self-limiting mechanism of TCR signaling. This energy-dependent regulatory mechanism complements the well-known extrinsic inhibitory axes mediated by PD-1, CTLA-4, and LAG-3 (Wherry and Kurachi, 2015), and resolves a longstanding puzzle why weak antigens (or chronic stimulation) induce only partial CD3ζ phosphorylation. While Germain’s group previously attributed this to continuous recruitment of phosphatases like SHP-1 (Madrenas et al., 1995; Stefanová et al., 2003), Xu’s study reveals a lipid- and energy-dependent alternative pathway that operates independently of inhibitory factors.

For immunotherapy, this finding suggests a new combinatorial strategy: pairing immune checkpoint blockade (ICB) with interventions that restore mitochondrial metabolism or boost ATP production. By reversing ATP depletion, such approaches could reinstate full CD3ζ phosphorylation, thereby revitalizing exhausted T cells and amplifying the efficacy of ICB therapy.

The BRS motifs identified in CD3ζ are not unique. Indeed, they are conserved in ∼70% of human single-pass transmembrane proteins, including key immune receptors such as B cell receptor (BCR), CD28, LAG3, and PD-L1 (Shi et al., 2025). This widespread conservation suggests that the BRS-lipid electrostatic interactions represent a general regulatory principle governing the functions of immune receptors. Membrane association of BRS acts as a molecular gatekeeper, shielding ITAMs from unwanted phosphorylation. Upon ligand engagement, BRS and ITAM regions are released from the membrane to fulfill ubiquitination, phase separation, and mechanotransduction (Chen et al., 2023; Jiang et al., 2025; Wu et al., 2019; Xu et al., 2008).

In light of prior studies report that the CD3ε subunit also exhibits membrane association and gradient phosphorylation properties (e.g., via conformational changes, receptor clustering, or altered membrane ionic environment), this work raises a fundamental question regarding TCR triggering: which subunit serves as the initial or primary sensor of antigen engagement— CD3ε or CD3ζ? Resolving this will require structural studies of the full TCR/CD3s complex including cytoplasmic domains in ligand-free and ligand-bound states. Nevertheless, the sequential phosphorylation hierarchy of CD3ζ provides a critical clue that antigen strength dictates “phosphorylation depth,” with membrane-proximal ITAM phosphorylation priming subsequent modification of membrane-distal sites.

## Conclusion

This study by Xu and colleagues represents a significant conceptual advance in TCR biology. By uncovering a lipid-regulated phosphorylation hierarchy within CD3ζ, it provides a unifying explanation for understanding how the TCR generates graded signals, why chronic stimulation induces T-cell exhaustion, and how membrane electrostatics fine-tune immune receptor activation. This work exemplifies the power of multidisciplinary approach by integrating structural biology (NMR), biochemistry (phosphorylation kinetics), and immunology (T cell exhaustion), which is increasingly essential for disentangling complex signaling mechanisms. How mechano-regulation along with molecular conformational changes and spatial organization intersect with this graded signaling model remains an intriguing question for future investigation.

Beyond its mechanistic insights, this study opens new avenues for therapeutic innovation. Potential strategies include engineering CAR-T cells with optimized BRS-ITAM sequences, developing small molecules to restore CD3ζ phosphorylation in exhausted T cells, and targeting BRS-lipid interactions to modulate signaling other immune receptors. As we continue to decipher the sophisticated molecular language of TCR signaling, this study brings us a step closer to precisely harnessing T cell immunity for treating cancer, infections, and autoimmune diseases.

**Figure 1.**
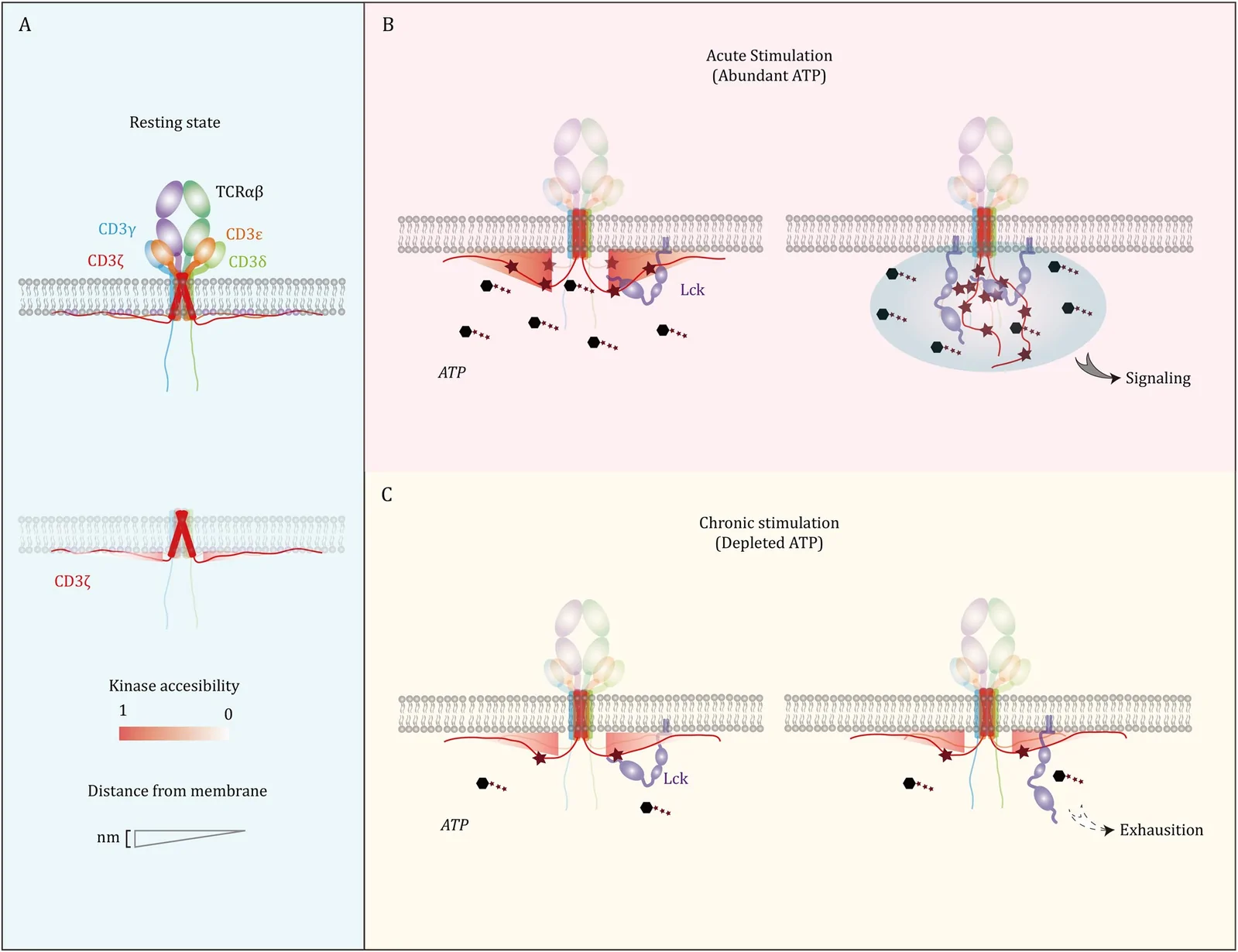
A model for TCR graded signaling and T-cell exhaustion regulated by a lipid-gated “phosphorylation code.” (A) In the resting state, the intracellular domains of CD3ε and CD3ξ are associated with the plasma cell membrane, restricting kinase accessibility. (B) During acute stimulation, increased kinase accessibility and high intracellular ATP levels promote downstream signaling. (C) Under chronic stimulation, reduced kinase accessibility and ATP depletion result in partial phosphorylation of CD3ξ, attenuated TCR signaling, and T-cell exhaustion.
